# Are three antiseptic paints needed for safe preparation of the surgical field? A prospective cohort study with 239 patients

**DOI:** 10.1186/s13756-020-00780-z

**Published:** 2020-07-31

**Authors:** Jan A. Roth, Cyrill Schwab, Andrew Atkinson, Markus von Flüe, Christoph Kettelhack, Friedrich S. Eckstein, Manuel Battegay, Steffi Klimke, Reno Frei, Andreas F. Widmer

**Affiliations:** 1 Division of Infectious Diseases and Hospital Epidemiology, University Hospital Basel, Petersgraben 4, 4031 Basel, Switzerland; 2 University of Basel, Basel, Switzerland; 3 Basel Institute for Clinical Epidemiology and Biostatistics, University Hospital Basel, Basel, Switzerland; 4 Department of Infectious Diseases and Hospital Epidemiology, University Hospital Bern, University of Bern, Bern, Switzerland; 5 Pediatric Pharmacology and Pharmacometrics Research, University Children’s Hospital Basel, Basel, Switzerland; 6 Clarunis, University Center for Gastrointestinal and Liver Disorders, University Hospital Basel, Basel, Switzerland; 7 Department of Cardiac Surgery, University Hospital Basel, Basel, Switzerland

**Keywords:** Antisepsis, Disinfection, Infection prevention, Method, Surgery

## Abstract

**Background:**

Preoperative skin antisepsis is an essential component of safe surgery. However, it is unclear how many antiseptic paints are needed to eliminate bacteria prior to incision. This study compared microbial skin counts after two and three antiseptic paints.

**Methods:**

We conducted a prospective cohort study in non-emergency patients receiving a cardiac/abdominal surgery with standardized, preoperative skin antisepsis consisting of an alcoholic compound and either povidone iodine (PI) or chlorhexidine (CHX). We obtained three skin swabs from the participant’s thorax/abdomen using a sterile template with a 25 cm^2^ window: After collection of the first swab prior to skin antisepsis, and once the second and third application of PI/CHX had dried out, we obtained a second and third swab, respectively. Our primary outcome was the reduction in microbial skin counts after two and three paints of PI/CHX.

**Results:**

Among the 239 enrolled patients, there was no significant difference in the reduction of mean square root-transformed microbial skin counts with three versus two paints (*P* = 0.2). But distributions of colony forming units (CFUs) decreased from paint 2 to 3 in a predefined analysis (*P* = 0.002). There was strong evidence of an increased proportion of patients with zero CFU after paint 3 versus paint 2 (*P* = 0.003). We did not identify risk factors for insufficient reduction of microbial skin counts after two paints, defined as the detection of > 5 CFUs and/or ≥ 1 pathogens.

**Conclusions:**

In non-emergency surgical patients, three antiseptic paints may be superior to two paints in reducing microbial skin colonization prior to surgery.

## Brief summary

This prospective cohort study indicated that in non-emergency surgery patients, three consecutive antiseptic paints may be superior to two antiseptic paints in reducing microbial skin counts prior to surgery.

## Introduction

Surgical site infections (SSIs) are associated with increased morbidity, mortality and healthcare costs [1, 2]. Most SSIs after elective surgery may relate to residual, viable bacteria at the surgical site; therefore, preoperative skin antisepsis is a cornerstone of SSI prevention, coupled with routine antimicrobial prophylaxis to avoid regrowth of residual bacteria [3–5]. However, skin antisepsis practices are heterogeneous across different healthcare institutions and countries. Although the antimicrobial effectiveness of preoperative surgical site preparations may depend on both the antiseptic agent used and its specific application method, it is still unclear how many antiseptic paints are needed to adequately reduce microbial skin colonization at the surgical site: As of yet, no international, evidence-based recommendations exist on this topic, and experimental standards to compare and license preoperative application techniques for skin antisepsis are not established [6, 7].

We therefore aimed to compare the effectiveness of two versus three antiseptic paints in reducing the microbial skin colonization at the surgical site. We hypothesized that three preoperative paints with either chlorhexidine (CHX) or povidone iodine (PI) are superior to two paints in reducing microbial skin counts.

## Methods

### Study design and setting

We performed a prospective cohort study at the University Hospital Basel (USB) ─ a tertiary care center in Switzerland with > 1700 abdominal and > 850 cardiac interventions per year (overall, ~ 38,000 surgical interventions per year). The present observational study was nested within an ongoing multicenter open-label cluster-randomized cross-over study on the efficacy of CHX versus PI skin antisepsis in preventing SSIs (i.e. PICASSo trial; ClinicalTrials.gov numbers, NCT03685604 and NCT03859375). The study data can be shared by the corresponding author upon request.

### Patient selection

At the USB, inpatients aged ≥18 years receiving a non-emergency cardiac or abdominal surgery between April 15, 2019 and September 06, 2019 were eligible for study inclusion. We excluded patients who could not provide or declined the written informed consent for the nested study. The PICASSo trial protocol does not include an individual informed consent.

### Study outcomes

Our predefined primary outcome was the reduction of microbial skin counts (that is, colony forming units [CFUs]) after two and three antiseptic paints, respectively. Secondary outcomes were (i) the proportion of patients with microbial skin counts of zero CFU after three antiseptic paints as compared to two antiseptic paints, and (ii) the proportion of patients with insufficient reduction of microbial skin counts after two antiseptic paints. We defined an insufficient reduction in microbial skin counts at the surgical site as the detection of > 5 CFUs and/or ≥ 1 pathogen(s) according to the National Healthcare Safety Network common commensals/pathogen list (version 9.2; www.cdc.gov). All outcomes were standardized per 25 cm^2^ skin area.

In a secondary analysis, we merged the prospectively collected SSI status within 30 days after surgery using a national SSI surveillance database [8, 9]. In this validated surveillance program, well-trained infection control practitioners ascertain SSIs by screening surgical patients for evidence of SSIs, and cases are double-checked by a board-certified infectious disease specialist. Standardized postdischarge SSI surveillance is conducted by telephone interviews and review of electronic medical records. SSIs are classified according to the Centers for Disease Control and Prevention definitions [10].

### Study procedures

Patients routinely receive a whole body shower with CHX (CHX digluconate 40 mg/ml solution; Hibiscrub®, Mölnlycke Health Care AG, Schlieren, Switzerland) prior to cardiac or abdominal surgery. Furthermore, it is standard practice to clip hair prior to surgery ─ if deemed necessary. Presurgical antiseptic processes and management are in accordance with the recommendations of the World Health Organization (WHO) [4]. At the USB, it is standard of care to consecutively perform skin antisepsis for three times (approximately 3 min at a time) by using sterile gauzes. The routinely applied skin antiseptics are either CHX in alcohol (CHX digluconate 20 mg and propan-2-ol 0.7 ml; Softasept® CHX, B. Braun Medical AG, Sempach, Switzerland) or PI in alcohol (PI 0.9 mg and propan-2-ol 457.5 mg; Braunoderm®, B. Braun Medical AG, Sempach, Switzerland). The PICASSo trial did not affect the routine procedures for skin antisepsis as recommended by the WHO [4], apart from the regular randomized department-level switches from presurgical skin antisepsis with PI to CHX, or vice versa.

For the present study, well-instructed members of the surgical team obtained the skin swabs in the operating room under sterile conditions. We collected three skin swabs (sterile 0.9% sodium chloride premoistened swabs; FLOQSwab®, Copan Diagnostics Inc., Brescia, Italy) from the participant’s trunk. To standardize the skin area, we used sterilized metal templates with a window of 25 cm^2^, which was swabbed repeatedly horizontally and vertically in a uniform way under gentle pressure. The template was freely positionable at the surgical site (thorax or abdomen) as long as it did not interfere with the succeeding incision. Following collection of the first skin swab prior to skin antisepsis, and once the second and third application of PI or CHX had dried out, we obtained a second and third skin swab, respectively. We did not collect swab samples at later time points, as the surgical incision may follow directly after the drying of the third application of PI or CHX (that is, start of the at-risk period).

The first skin swab was neutralized for CHX to avoid bias by presurgical shower with CHX. Swabs 2 and 3 were neutralized before culture for either CHX or PI ─ depending on the applied antiseptic product. We used a standardized inactivation solution, which was tested for non-toxicity and which was microbiologically validated ─ containing either polysorbate 80 30 g/l, lecithin 3 g/l, L-histidine 1 g/l, sodium thiosulfate 5 g/l, saponine 30 g/l, trypticase soy broth 30 g/l, and distilled water 1 l for CHX skin antisepsis; or polysorbate 80 30 g/l, lecithin 3 g/l, L-histidine 1 g/l, sodium thiosulfate 5 g/l, trypticase soy broth 30 g/l, and distilled water 1 l for PI skin antisepsis (details can be requested from the corresponding author).

### Data collection

A study physician collected the relevant clinical information during the screening visit and verified the respective data by use of electronic medical records. In the operation room, a trained study nurse recorded the applied antiseptic product and timing of skin swab collection.

### Microbiological investigation

Skin swabs were immediately delivered to the in-house microbiological laboratory, where a study technician inoculated trypticase soy agar (TSA) plates (bioMérieux, Marcy l’Etoile, France) within 6 h during weekdays or within 72 h on weekends (samples were kept in the refrigerator before processing). Due to logistical reasons, we could not mask the study technician for the exposure status (that is, consecutive paint number; 1 to 3). We cultured an additional 1:10 sodium chloride diluted sample of the first swab to safeguard against unreliable results for samples with high microbial counts. We incubated the TSA plates for 2 days at 36 °C (±1 °C) and determined respective CFUs with a manual colony counter (Scan® 100, Intersciences, Saint Nom, France). We identified microbial skin isolates using the microflex™ LT MALDI-TOF mass-spectrometer system (Bruker Daltonics, Bremen, Germany).

### Statistics

Based on a previous internal quality evaluation (unpublished data), we estimated a sample size of ≥228 patients in order to demonstrate superiority of three versus two paints at a clinically defined superiority margin of 2 log_10_(CFUs) difference (significance level of 5%, power of 90%). Our null hypothesis was that there was no difference between two and three paints of PI/CHX in reducing microbial skin counts (CFUs) at the surgical site.

With regards to the primary outcome, we compared the square root-transformed CFUs after paint 2 and 3 using a paired t-test. In a supplementary analysis of the primary outcome, we also compared the CFU distribution after paints 2 and 3 using the Wilcoxon signed rank test. We compared secondary outcomes (proportions) after paint 2 and 3 using a χ^2^-test (overall and stratified by PI/CHX application). In a secondary analysis, we compared CFU counts after paint 3 between patients with and without a subsequent SSI (within 30 days after index surgery) using the Wilcoxon rank sum test.

We fitted univariable logistic regression models (variables and categories provided in Table 3) to identify potential risk factors for insufficient reduction of microbial skin counts after paint 2. A low event-predictor ratio precluded a multivariable logistic regressions analysis. Results were considered significant at a *P*-value of ≤0.05. Analyses of secondary outcomes and subgroups were considered as hypothesis-generating. The study data were analyzed by a statistician (A.A.) using the R statistical software (www.r-project.org).

## Results

**Fig. 1:**
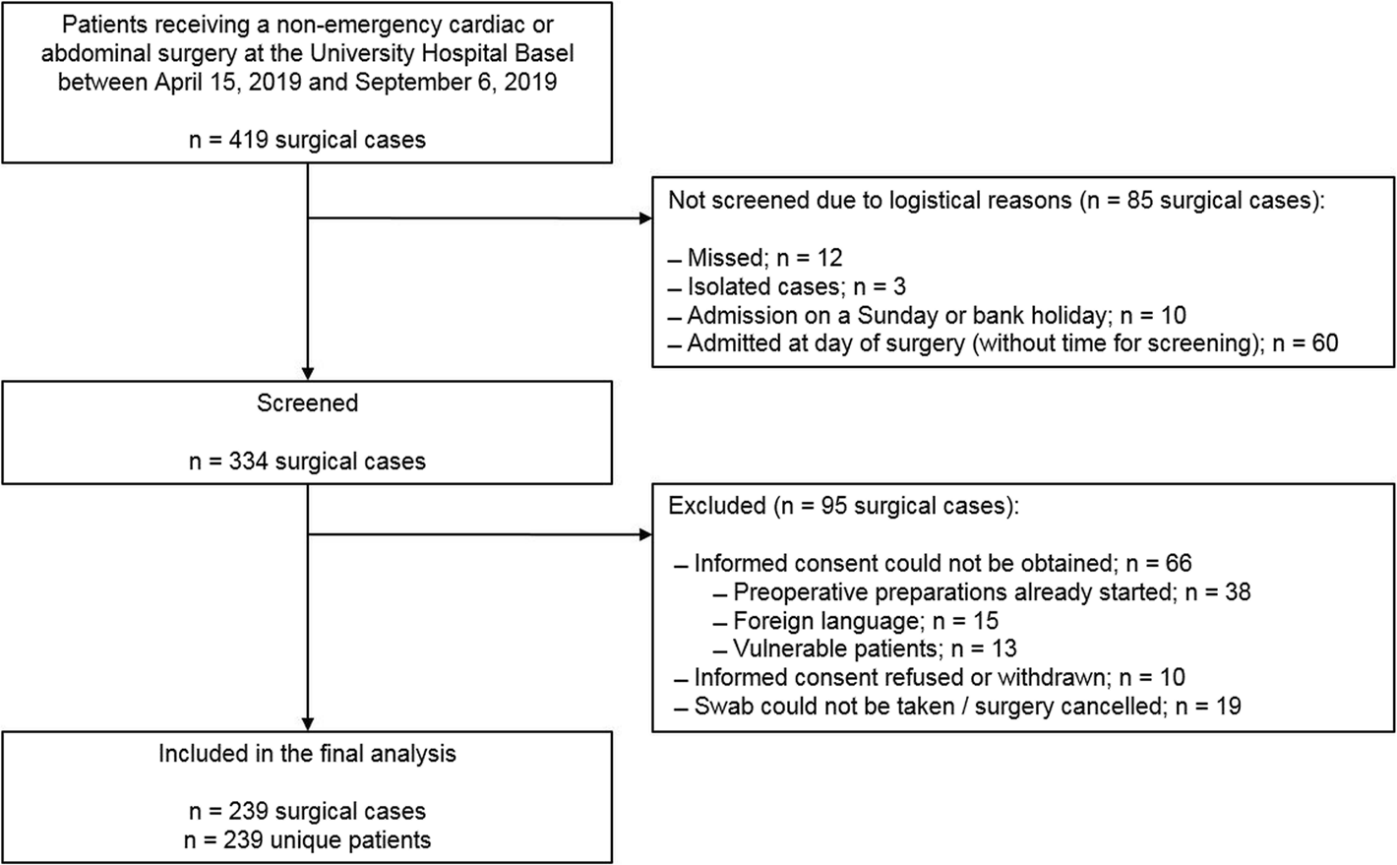
Patient Selection

During the study period, we included 239 of 334 (71.6%) screened patients who received a non-emergency cardiac or abdominal surgery (Fig. 1); we excluded the remaining 95 screened patients due to logistic reasons or refused/withdrawn informed consents.

Overall, the median age of the study population was 65.0 years (interquartile range [IQR], 56.5 to 72.5 years) and 231 of 239 patients (96.7%) received an adequate antimicrobial prophylaxis (Table 1). Due to ongoing cluster randomization, 94 and 145 of 239 patients (39.3 and 60.7%) received preoperative skin antisepsis with CHX and PI, respectively. The median overall PI/CHX exposure time was 7.6 min (IQR, 6.5 to 9.0 min). Fifteen of 239 (6.3%) patients developed an SSI within 30 days after surgery.

**Table 1:** Baseline characteristics of patients undergoing a non-emergency cardiac or abdominal surgery (*n* = 239 patients)

Characteristics	Overall	No. patients with missing data^**a**^
Age in years,^b^ median (IQR)	65.0 (56.5─72.5)	0
Female, n (%)	75 (31.4)	0
BMI in kg/m^2^,^b^ median (IQR)	26.8 (23.8─30.1)	0
Diabetes mellitus,^c^ n (%)	48 (20.1)	0
Antimicrobial treatment prior to surgery,^d^ n (%)	15 (6.3)	0
Adequate antimicrobial prophylaxis,^e^ n (%)	231 (96.7)	0
Type of surgery, n (%)		0
Cardiac surgery	135 (56.5)	
Abdominal surgery	104 (43.5)	
Antiseptic product,^f^ n (%)		0
Chlorhexidine	94 (39.3)	
Povidone iodine	145 (60.7)	
Overall exposure time of antiseptics^g^ in minutes, median (IQR)	7.6 (6.5─9.0)	4

*BMI* body mass index, *IQR* interquartile range

^a^For each row/variable

^b^At day of surgery

^c^Previous diagnosis of diabetes mellitus type 2 according to medical records

^d^Any antimicrobial treatment within the last 2 weeks prior to surgery and excluding peri-interventional antimicrobial prophylaxis

^e^Antimicrobial prophylaxis administered within 120 min prior to incision

^f^The formulations in use for preoperative skin antisepsis were chlorhexidine in alcohol and povidone iodine in alcohol

^g^Time period from start of skin antisepsis until the applied antiseptic has dried out after paint 3

**Fig. 2:**
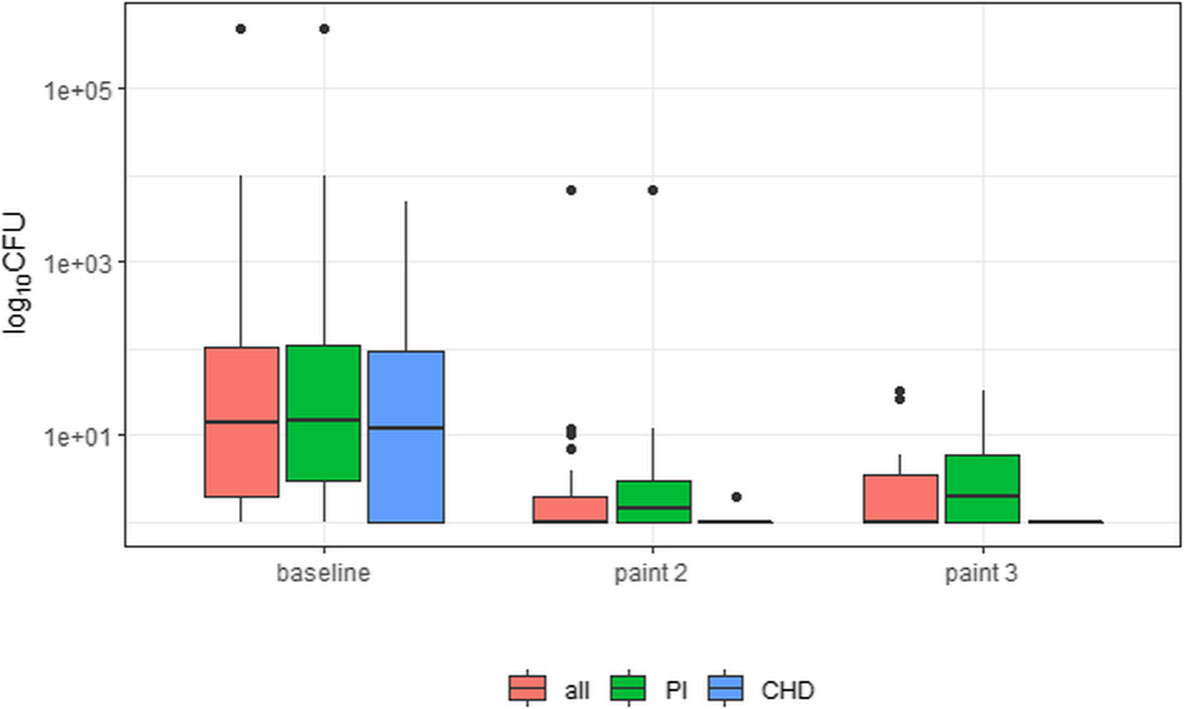
Microbial skin counts; overall and stratified by antiseptic product. CFU, colony forming units; CHD, chlorhexidine; PI, povidone iodine. Note: Among the 239 individuals, CFU data were missing for 1 patient at baseline, for 3 patients after paint 2, and for 2 patients after paint 3. Regarding the log-transformed CFU values, boxes cover the median and 25 to 75% percentiles. CFU counts were increased by a very small fraction to avoid zero values

Regarding the primary outcome, there was overall no statistically different reduction in square root-transformed microbial skin counts with three versus two paints (*P* = 0.2; Table 2); but there was strong evidence in a supplementary analysis that the respective CFU distributions were different for paint 2 and 3 (*P* = 0.002): This difference could also be observed in the PI subgroup but not in the CHX subgroup. Median CFUs after paint 2 and 3 were 0.0 (IQR, 0.0 to 0.0) in the overall study population and the PI and CHX subgroups, respectively. For illustration purposes, we depict the overall and PI/CHX-stratified CFU counts in Fig. 2.

**Table 2:** Microbial skin counts prior to and during preoperative skin antisepsis

Microbial skin counts/pathogens^a^	Timing			*P*-value for difference (paint 2 vs. 3)
	Prior to skin antisepsis^b^	After 2nd paint	After 3rd paint	
Overall				
$\sqrt{\mathrm{CFU}},$ mean (SD)	11.2 (47.5)	0.5 (5.5)	0.1 (0.6)	0.2
CFU, median	7.0	0.0	0.0	0.002
CFU, IQR	1.0 to 79.8	0.0 to 0.0	0.0 to 0.0	
CFU, range	0.0 to 50,000.0	0.0 to 7000.0	0.0 to 33.0	
No. patients with 0 CFU (%)	30 (12.6)	203 (86.0)	224 (94.5)	0.003
No. patients with > 5 CFU (%)	126 (52.9)	5 (2.1)	3 (1.3)	0.9
No. patients with ≥1 pathogens^c^ detected (%)	1 (0.4)	1 (0.4)	0 (0)	0.5
No. patients with insufficient microbial reduction^a,d^ (%)	0 (0)	6 (2.5)	3 (1.3)	0.5
Povidone iodine subgroup				
$\sqrt{\mathrm{CFU}},$ mean (SD)	13.7 (60.2)	0.9 (7.0)	0.2 (0.72)	0.2
CFU, median	8.5	0.0	0.0	0.002
CFU, IQR	1.0 to 84.0	0.0 to 0.0	0.0 to 0.0	
CFU, range	0.0 to 50,000.0	0.0 to 7000.0	0.0 to 33.0	
No. patients with 0 CFU (%)	18 (12.5)	114 (80.2)	133 (93.0)	0.002
No. patients with > 5 CFU (%)	80 (55.2)	5 (3.5)	3 (2.1)	0.7
No. patients with ≥1 pathogens^c^ detected (%)	1 (0.7)	1 (0.7)	0 (0)	0.7
No. patients with insufficient microbial reduction^a,d^ (%)	0 (0)	6 (4.2)	3 (2.1)	0.5
Chlorhexidine subgroup				
$\sqrt{\mathrm{CFU}},$ mean (SD)	7.5 (12.4)	0.1 (0.3)	0.03 (0.2)	0.4
CFU, median	5.5	0.0	0.0	0.5
CFU, IQR	1.0 to 60.0	0.0 to 0.0	0.0 to 0.0	
CFU, range	0.0 to 5000.0	0.0 to 2.0	0.0 to 1.0	
No. patients with 0 CFU (%)	12 (12.8)	89 (95.0)	91 (97.0)	0.7
No. patients with > 5 CFU (%)	46 (48.9)	0 (0)	0 (0)	─
No. patients with ≥1 pathogens^c^ detected (%)	0 (0)	0 (0)	0 (0)	─
No. patients with insufficient microbial reduction^a,d^ (%)	0 (0)	0 (0)	0 (0)	─

Among the 239 individuals, CFU data were overall missing for 1 patient at baseline, for 3 patients after paint 2, and for 2 patients after paint 3

*CFU* colony forming units, *IQR* interquartile range, *SD* standard deviation

^a^In the antiseptic area (template window area, 25 cm^2^)

^b^Immediately before preoperative skin antisepsis (first paint)

^c^Pathogen defined according to the National Healthcare Safety Network common commensals/pathogen list (version 9.2; www.cdc.gov)

^d^Insufficient reduction was defined as detection of > 5 colony forming units and/or ≥ 1 pathogens in the examined, antiseptic area

Concerning the secondary outcomes, there was overall strong evidence of an increased proportion of patients with zero CFU after paint 3 versus 2 (94.5% versus 86.0%, *P* = 0.003; Table 2): This difference could be observed correspondingly in the PI subgroup but not in the CHX subgroup. The overall proportion of patients with insufficient reduction after paint 2 and 3 was similar (Table 2), but we were unable to identify risk factors for insufficient microbial reduction after two paints of PI/CHX (Table 3). Following paint 2, we isolated a pathogen in one of the 239 patient (that is, *Staphylococcus aureus*); after paint 3, no pathogens were detected in any of the 239 studied patients. In a secondary analysis, median CFU counts after paint 3 were similar for patients with and without subsequent SSI within 30 days after surgery (median CFU count, 0; IQR, 0 to 0; and median CFU count 0; IQR, 0 to 0; *P* = 0.8).

**Table 3:** Risk factors for insufficient reduction of microbial skin counts after two paints of preoperative skin antisepsis

Variable	Level	Crude^a^ OR	Crude^a^*P*-value
Age^b^	Per 1-year increase	1.0 (1.0─1.1)	0.3
Sex	Female	1	0.2
	Male	0.3 (0.04─1.8)	
BMI^b^	Per 1-kg/m^2^ increase	1.0 (0.8─1.2)	0.2
Diabetes mellitus^c^	No	1	0.3
	Yes	2.6 (0.3─16.4)	
Antimicrobial treatment prior to surgery^d^	No	1	0.2
	Yes	3.8 (0.2─28.0)	
Adequate antimicrobial prophylaxis^e^	No	1	Not estimable
	Yes	─	
Type of surgery	Cardiac	1	0.9
	Abdominal	0.8 (0.1─5.4)	
Type of disinfectant	Chlorhexidine	1	Not estimable
	Povidone iodine	─	
Overall exposure time of antiseptic product^f^	Per 0.5-min increase	1.1 (0.9─1.3)	0.6
Microbial skin counts prior to skin antisepsis	Per 100-CFU increase	1.0 (1.0─1.0)	0.9

We performed all univariable analyses on the complete case population (*n* = 230 patients)

*BMI* body mass index, *CFU* colony forming unit, *OR* odds ratio

^a^Calculated by use of univariable logistic regression models with fixed effects

^b^At day of surgery

^c^Previous diagnosis of diabetes mellitus type 2 according to medical records

^d^Any antimicrobial treatment within the last 2 weeks prior to surgery and excluding peri-interventional antimicrobial prophylaxis

^e^Antimicrobial prophylaxis administered within 120 min prior to incision

^f^Time period from start of skin antisepsis until the applied antiseptic has dried out after paint 2

## Discussion

In contrast to hand hygiene and other infection prevention measures, evaluations of preoperative techniques for skin antisepsis might be considered a neglected research area ─ despite its potential impact on morbidity, mortality, logistics and healthcare costs [6, 7, 11–13]. In the present prospective cohort study performed in non-emergency patients receiving a cardiac or abdominal surgery, we observed overall similar square root-transformed microbial skin counts after two and three applications of PI or CHX. In a supplementary analysis on untransformed microbial skin counts, there was evidence, however, of different CFU distributions after three versus two paints ─ in both the overall study population and the PI subgroup. Furthermore, there was overall strong evidence of an increased proportion of patient with zero CFU after paint 3 versus 2. To our knowledge, this is the first clinical study that compared the antimicrobial effectiveness of different numbers of PI/CHX applications for preoperative skin antisepsis.

The somewhat conflicting results of our primary and supplementary analysis may be explained by the different nature of the two test procedures (comparison of mean CFUs and the shape of CFU distributions, respectively) with the former test statistic requiring approximately equal variance between groups: This was not the case in our sample, even with appropriate data transformations. In the PI and CHX subgroup, the observed differential effect in the reduction of microbial skin counts after paint 2 and 3 (Table 2) could be related to an improved antimicrobial effectiveness of CHX [4, 5]. As we had collected CHX-inactivated microbial skin swabs directly after paint 2 and 3, it seems unlikely that prolonged antimicrobial effects of CHX resulted in the observed differential effect.

Interestingly, we did not identify potential risk factors for insufficient microbial reduction after two paints of CHX or PI (Table 3). This may indicate that unmeasured variables and/or non-estimable associations in strata without cases (e.g. patients who received adequate antimicrobial prophylaxis) may contribute to a differential reduction in microbial skin counts after two paints of CHX or PI. Currently, this observation may preclude application of only two antiseptic paints in possible low-risk surgical subgroups.

Our study has strengths. Firstly, we conducted an adequately powered prospective cohort study with consecutive PI/CHX inactivation of swab samples using validated inactivation solutions: Previous studies assessing the antimicrobial effectiveness of different skin antiseptics may have frequently been based on swab samples, which were not PI/CHX inactivated: This could lead to outcome misclassification. Secondly, we chose a pragmatic study approach that may depict the antimicrobial effectiveness of skin antisepsis techniques under real-world conditions. Thirdly, our study was conducted by a well-instructed study team limiting the potential for information bias.

Nonetheless, our study has limitations. Firstly, our study was not powered to detect differences in the antimicrobial effectiveness (swab 2 versus 3) for the given subgroups. These stratified analyses should be considered as hypothesis-generating only. Secondly, due to logistical reasons, we could not mask our study technician for the exposure status (that is, paint number). However, it is highly unlikely that the known exposure status has led to systematic errors when counting microbial skin counts. Thirdly, we cannot exclude the possibility that exclusions of patients due to refused informed consents and logistical reasons may have resulted in selection bias. Nevertheless, it is unlikely that excluded patients would show differential antimicrobial effects after two versus three paints, as reasons for exclusion were manifold and probably not related to the propensity for inadequate microbial reduction. As we analyzed intra-subject differences in microbial skin counts, we have accounted for static confounders in the paint number-CFU relationship. Fourthly, our sample size and the institution-wide recommendation to apply three preoperative antiseptic paints precluded the investigation of the paint number-SSI relationship. Lastly, our study was performed in non-emergency surgical patients under a study setting ─ potentially leading to a Hawthorne effect: Our results may not be generalizable to other surgical populations and settings.

## Conclusion

In non-emergency surgical patients, three consecutive antiseptic paints may be superior to two paints in reducing microbial skin colonization prior to surgery. Our observational findings warrant further evaluation in a randomized trial to better characterize the clinical efficacy and effectiveness of different skin antisepsis methods. Evidence-based standards on preoperative techniques for skin antisepsis are needed.

## Data Availability

Not applicable.

## Abbreviations

BMI: Body mass index
CI: Confidence interval
CFU: Colony forming unit
CHX: Chlorhexidine
IQR: Interquartile range
OR: Odds ratio
PI: Povidone iodine
SD: Standard deviation
SSI: Surgical site infection
TSA: Trypticase soy agar
USB: University Hospital Basel
